# Framework for Health-Promoting Environments for Office Workers: Photovoice Study

**DOI:** 10.2196/90712

**Published:** 2026-06-29

**Authors:** Ulrika Florin, Christina Bodin Danielsson, Katarina Bälter, Susanna Lehtinen-Jacks

**Affiliations:** 1 Department of Health Sciences, Innovation and Design, Faculty of Engineering and Health Sciences, Mälardalen University, Box 883, Västerås, 721 23, Sweden, 46 21101478; 2 Architecture, Technique and Theory, School of Architecture, KTH Royal Institute of Technology, Stockholm, Sweden; 3 Department of Construction Management, School of Architecture and Civil Engineering, Chalmers University of Technology, Gothenburg, Sweden; 4 Department of Medical Epidemiology and Biostatistics, Karolinska Institutet, Stockholm, Sweden

**Keywords:** prevention and health promotion, employee health, office work environment, remote work, flexible work arrangements, public health research, photovoice, visual methods, user-involving research, digital data collection, visual framework, questionnaire development, salutogenesis, environmental aspects, behavioral aspects, Sweden

## Abstract

**Background:**

Office work is increasingly carried out outside conventional office settings, particularly during and after the COVID-19 pandemic. This highlights the need to understand the complexity of aspects that may influence health across different office work environments.

**Objective:**

This study aimed to (1) identify aspects that office workers perceive as supporting or hindering their health during office work, and (2) formulate novel questions about office work for quantitative studies.

**Methods:**

In February 2021, we conducted a digitally distributed photovoice study in Sweden, in which a convenience sample of 17 office workers from 5 companies took photos and provided written comments on what they perceived as supporting or hindering their health in places where they performed office work. For objective 1, we carried out both qualitative formal analysis and analysis without a theoretical frame, as well as quantified the content of the photos and comments. The identified aspects and their interactions were summarized in a visual framework. For objective 2, findings from the photovoice study were used to adapt selected items from the 2019 Swedish Work Environment Survey, capturing office work performed across multiple settings.

**Results:**

Of a total of 63 photos, 70% (44/63) were taken at home, 24% (15/63) in an office, and 6% (4/63) outdoors. The comments on photos taken in conventional office settings largely highlighted health-promoting aspects, while the interpretations of home office photos showed greater variability regarding their impact on health. We identified 9 aspects and categorized them into two groups: (1) environmental perspective, including space, ergonomic, technical, and aesthetic-sensuous aspects and (2) behavioral perspective, including flexibility, focus, breaks-recovery, physical activity, and eating habits. Whether the aspects supported or hindered health depended on the environment where office work was performed and the employees’ living conditions. Our visual framework illustrates how these two perspectives interact with each other, bridged by space and flexibility. The study also resulted in a battery of multiple-choice questions about work in offices, at home, in public places, and outdoors that can be used in future research to better capture variation in modern work arrangements.

**Conclusions:**

This study extends the notion that office environments play a central role in supporting employee health by suggesting that this also applies to home offices. The results emphasize the need for tailored health-promoting interventions that account for the diverse environments in which office work is performed and for individual needs. The developed visual framework for analyzing health-promoting work environments for office workers and the battery of survey questions can contribute to future research and the advancement of sustainable, health-promoting office environments.

## Introduction

International health organizations highlight workplace health promotion as an integrated part of occupational health [1]. This aligns with Stokols socioecological model of health, emphasizing interactions between individual and contextual determinants for promoting healthy lifestyles [2]. In workplace design research, Roskams and Haynes [3] have proposed a conceptual framework for salutogenic workplace design that focuses on supporting a sense of coherence through environmental resources. Yet, there is limited empirical research that identifies and integrates health-promoting (salutogenic) resources relevant to contemporary office work [3,4].

This study focuses on factors that may promote or hinder health during office work, since office work is common; for example, around 60% of the workforce in Sweden performs office work at least a quarter of their working time [5]. Office work tends to contain a lot of sedentary time [6-9] and can involve barriers to healthy eating [10], making health-promoting resources relevant to consider in the contexts where office work is performed.

Recent research links office design and specific workplace features to employees’ health and well-being [4,11-14]. This research provides important insights, including that interactions between spatial attributes may matter for outcomes such as comfort and connectedness [14]. Further, a study suggests that overall well-being may differ between home and office settings, and that specific aspects of well-being may be rated higher in one setting than the other [12]. However, much of this evidence base has focused on predefined spatial attributes [4,12,14] and often uses experimental designs [4,14].

In parallel, office work is increasingly performed outside conventional office settings, particularly during and after the COVID-19 pandemic [15]. Research on working from home has shown that experiences may be perceived as positive or challenging depending on a range of factors [11,16], including employment characteristics, gender, isolation and loneliness, co-worker support, leadership style and support, and home office conditions [11].

These developments and findings can be understood within a broader transformation in how office work is organized—often discussed under the umbrella of new ways of working (NWW). NWW is a widely adopted human resources approach that, enabled by digitalization, aims to increase flexibility and autonomy for knowledge workers, including greater freedom to choose where and when to work [17]. As a result, the health implications of office work can no longer be assumed to be limited to the conventional office setting. Due to lessons learned from the COVID-19 pandemic, employees’ health has also been highlighted even more as an important measure of how well an organization supports its employees [18]. Although NWW may improve work-life balance and well-being at work, contradictory findings have been reported, and it is not yet fully understood how NWW affects the well-being of employees and organizations [17].

Aiming to better understand the effects that different environments for office work have on health, both qualitative and quantitative research are needed to provide both a broad and in-depth perspective. Existing quantitative epidemiological studies have traditionally focused on office work performed within the physical office setting. However, the increasing diversity of work environments calls for updated measures that also capture office work performed beyond the traditional office.

Against this background, the objectives of this study were twofold: (1) to identify aspects that office workers perceive as supporting or hindering their health during office work, using a photovoice study and (2) to use the photovoice data to formulate novel questions for quantitative studies on office work.

## Methods

### Study Setting and Design

This study was part of the large research project Concepts for the Sustainable Office of the Future (SOFCO) and was conducted within subproject 3, lifestyle behaviors, working conditions, and health among office workers. SOFCO aimed to promote healthy working lives and lifestyles and support sustainable office development. It involved working together with various companies from the office sector, including those that own or develop office buildings, provide shared workspaces, specialize in interior design, offer sustainable solutions, or consult on office-related matters. We used a qualitative research design with digital photovoice as the data collection method.

### Participants

We enrolled a convenience sample of office workers from 7 partner companies in the SOFCO project. Each partner company nominated a project leader (contact person). We asked them to recruit 4‐5 participants by sending email invitations to employees and stated our aim of recruiting participants with different positions at various levels within the company. The contact person was also allowed to participate. The invitation consisted of an information letter, an informed consent form, and practical instructions. We approached the contact persons 3 times between February 1, 2021, and February 15, 2021.

Eighteen persons (15 women and 3 men) from 5 companies gave their informed consent. Our final analytic sample consisted of 17 participants (14 women and 3 men) who provided photos and comments. Fourteen participants disclosed their job title, representing a diverse range of professional levels, primarily mid- to senior-level positions (eg, executive, managerial, specialist, and project leadership roles), as well as a few early-career positions. They held positions as Chief Executive Officer, Senior Consultant, Science and Marketing Specialist, Service Operative Manager, Sales Manager, Project Leader, Project Developer, Project Tenant, Project Leader Trainee, and Salesperson.

### Data Collection

The central method in this study was a variation of photo-elicitation often termed photovoice [19-23]. Through this method, participants shared their gaze on places, objects, and phenomena in their work environments and gave voice to the photos via short written comments. The main advantages of this method are that it allows data to be collected on how people interact with and within their environment and with things, while also revealing insights into how they feel about them, without interfering with their daily activities and schedules [19-21]. During the COVID-19 pandemic lockdown, such a method allowed participants and researchers to interact digitally without physical contact.

The invitation, including practical instructions, was sent to the potential participants via email by the contact person at each company. In the practical instructions, we asked the participants to take 3‐5 photos each, using their cell phone cameras, that showed or could be associated with anything they considered to be supporting or hindering health in their workplace and its surroundings, at home (remote work), or in other places where they performed office work. We also asked them to attach a short comment (approximately 1‐3 sentences) to each photo, supplementing its content or subjective meaning. The only restrictive instruction given to the participants was not to take identifiable photos of other people (ie, no facial images). A key difference compared to previous literature is that participants were not restricted to specific single or combined attributes. Instead, they were encouraged to document “anything” they perceived as promoting or hindering health in office work. This approach reduces the risk that the researcher’s selection of attributes in advance defines what appears relevant and makes it possible to capture aspects that might otherwise fall outside experimental designs.

Finally, the participants were asked to submit their photos and comments via email, no later than February 18, 2021. This date was set to allow sufficient time before the deadline for submitting the new battery of questions to the LifeGene project. One reminder was sent to the contact persons on February 15, 2021. We did not reach out to the participants for clarification or additional details.

### Analysis

**Figure 1.**
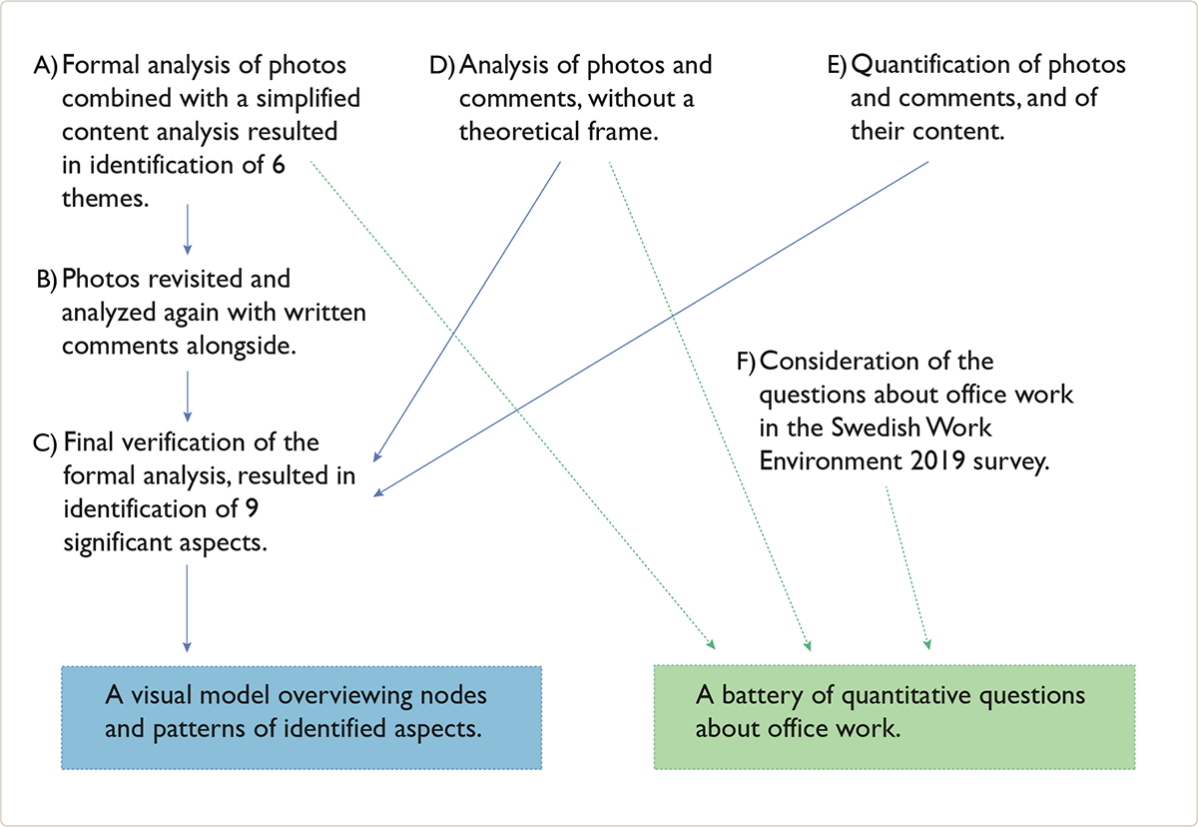
A schematic overview of the methods (steps A-F) and the workflow, represented by solid blue and dashed green arrows, leading to the resulting outputs shown in the blue and green boxes, respectively.

An overview of the analyses, together with the resulting outputs, is depicted in Figure 1.

First, we conducted a formal analysis together with a simplified qualitative content analysis to identify where the photos were taken, how they were composed, and what elements and objects they contained [24-26], marked as step A in Figure 1.

Formal analysis, as a methodological approach, has its origins in art history and can be traced back to formalist scholars such as Heinrich Wölfflin (1915/1950) [27] and to Erwin Panofsky preiconographical analysis of artworks (1939/1955) [28]. However, formal analysis is nowadays applied to all types of visual materials and has been further developed in fields such as visual studies, information design, and architecture, where it has also gained a more practical use in different design processes. The formal analysis is restricted to what can be directly seen in visual material (photos in this case) without interpreting symbolic meaning, narrative content, or cultural significance. In this formal analysis, we looked at colors, shapes, and forms, light and shadow, spatial organization and perspective, and placement of things. The content analysis specified what kinds of things there were [20].

We identified the following six themes in the photo sample and documented them as content descriptions for each photo: (1) office or home environment, (2) indoors or outdoors, (3) light and color aspects, (4) objects in the environment, (5) placement and use of objects, and (6) technological and social aspects of objects. Next, we revisited and analyzed the photos alongside participants’ comments to determine whether the comments contributed to altered interpretations of the photos [20]; step B in Figure 1. We verified the formal analysis by clustering photos and comments according to identified recurring patterns [20,29]; step C in Figure 1. From these clusters, we selected representative photos and quotations that illustrated 9 significant aspects. The quotes were translated from Swedish into English, except for those originally written in English by non-Swedish-speaking participants. The main actor in the formal analysis was a researcher with expertise in information design. In parallel with the formal analysis, a researcher with expertise in public health science carried out an analysis without a theoretical frame; step D in Figure 1. These results contributed to contextualizing the results from the formal analysis within the public health domain.

We also quantified the photos and comments based on where the photos were taken (office, home, or outdoor environment) and whether the comments included positive interpretations (supporting health), negative interpretations (hindering health), or both [20]; step E in Figure 1. We performed these quantifications per photo and accompanying comment, as well as per participant.

Finally, we developed a visual framework to provide an overview of the relationships between the 9 aspects. The main analyses were carried out between March 2021 and November 2022.

In addition, there were 2 more iterations, in the format of SOFCO annual workshops. During the first one (November 2021), the workshop participants were sent to breakout rooms for 20‐25 minutes to discuss a selection of 27 photos (from the total of 63). A panel discussion was held afterward, consisting of 1 representative from each partner company, on the question “What impressions and reflections did you get from the photovoice study?” In a final workshop (November 2024), the developed visual framework was discussed with the participating companies’ representatives to explore and capture their understanding.

### Formulating Novel Questions About Office Work

To achieve the second objective, we used the findings from the preliminary formal analysis and the analysis without a theoretical frame of the photovoice study to adapt specific items from the 2019 Swedish Work Environment Survey [30] to develop new questions related to office work, marked as steps A, B, and F in Figure 1. We applied and slightly revised 4 questions from the 2019 Swedish Work Environment Survey [30] related to working time in an office environment, type of office environment, access to a room for specific tasks that may require silence, and experience of whether the office environment allows one to do a good job (respective question numbers 65, 66, 67, and 69), as described in the Results section. Based on the results from the photovoice study, we added new symmetrical questions to address other environments for office work. To test the new battery of questions, 10 of the photovoice participants from 5 companies were invited to a pilot study; 5 of them decided to participate. Based on the feedback, one answer option, “Most often 5 days a week,” was changed to “5 days a week or more.” The new questions, now integrated into the large population-based Swedish LifeGene study [31,32], are described in the Results section.

### Ethical Considerations

Subproject 3 within SOFCO has been approved by the Swedish Ethical Review Authority (case 2021‐02549). We conducted all study procedures in accordance with the Swedish Act (2003:460) concerning the ethical review of research involving humans [33], institutional guidelines [34,35], and observed the privacy rights of human participants throughout the research process. Participants did not receive any compensation. All participants provided their written informed consent to participate in this study.

## Results

### Characteristics of Photos and Written Comments

The 17 participants from 5 companies submitted a total of 63 photos with written comments (Table 1). The number of photos varied between 2 and 6 per participant. Each participant submitted 1 comment per photo, except for 3 participants who wrote a single comment that addressed their 2 or 3 photos together. The comments were most often 1-3 sentences long (as instructed), while some participants wrote longer comments; the longest one consisted of 16 sentences. Accordingly, some participants pointed out 1 factor in the respective photo that they considered supporting or hindering their health; while some participants thoroughly explained multiple items in 1 photo.

Of the photos, 70% (44/63) were taken in home environments (Table 2). When examined within each environment, the comments on photos taken at conventional office settings largely highlighted health-promoting aspects (73%, 11/15), while the interpretations of home photos showed greater variability, including comments categorized as supporting health (34%, 15/44), hindering health (25%, 11/44), or both (41%, 18/44).

**Table 1.** Information about the participants and the received data, per company and in total.

Characteristic	Company: A	Company: B	Company: C	Company: D	Company: E	Total
Participants, n (%)	2 (12)	3 (18)	6 (35)	5 (29)	1 (6)	17 (100)
Photos, n (%)^a^	7 (11)	11 (17)	20 (32)	20 (32)	5 (8)	63 (100)
Photos per participant (min), n^a^	3	3	2	3	5	2
Photos per participant (max), n^a^	4	5	6	6	5	6

a Photos and the related written comments.

**Table 2.** Distribution of the interpretations (supporting or hindering health, or both) in the comment, by location where the photo was taken (in an office, at home, or outdoors).^a^

Photo location	Supporting health, n (%)^b^	Hindering health, n (%)^b^	Both, n (%)^b^	Total, n (%)^b^
Photo taken				
In an office	11 (17)	4 (20)	0 (0)	15 (24)
At home	15 (24)	11 (17)	18 (29)	44 (70)
Outdoors	2 (3)	0 (0)	2 (3)	4 (6)
Total, n (%)^b^	28 (44)	15 (24)	20 (32)	63 (100)

a In addition, one person sent a separate comment that connotes hindering health (not connected to any specific photo).

b All percentages are calculated out of the total number of photos with accompanying comments (N=63), not by row or column.

### Aspects Supporting or Hindering Health

#### Overview

Results from the initial formal analysis, the first SOFCO workshop, and the revisiting of the photos together with the respective written comments are shown in Multimedia Appendix 1. The final verification of the formal analysis led to the identification of aspects related to what participants considered to be supporting or hindering their health at any place where they performed office work. We identified 9 significant aspects: spaces, physical ergonomics, technical, aesthetic-sensuous, flexibility, focus, breaks-recovery, physical activity, and eating habits*.*

#### Spaces

**Figure 2.**
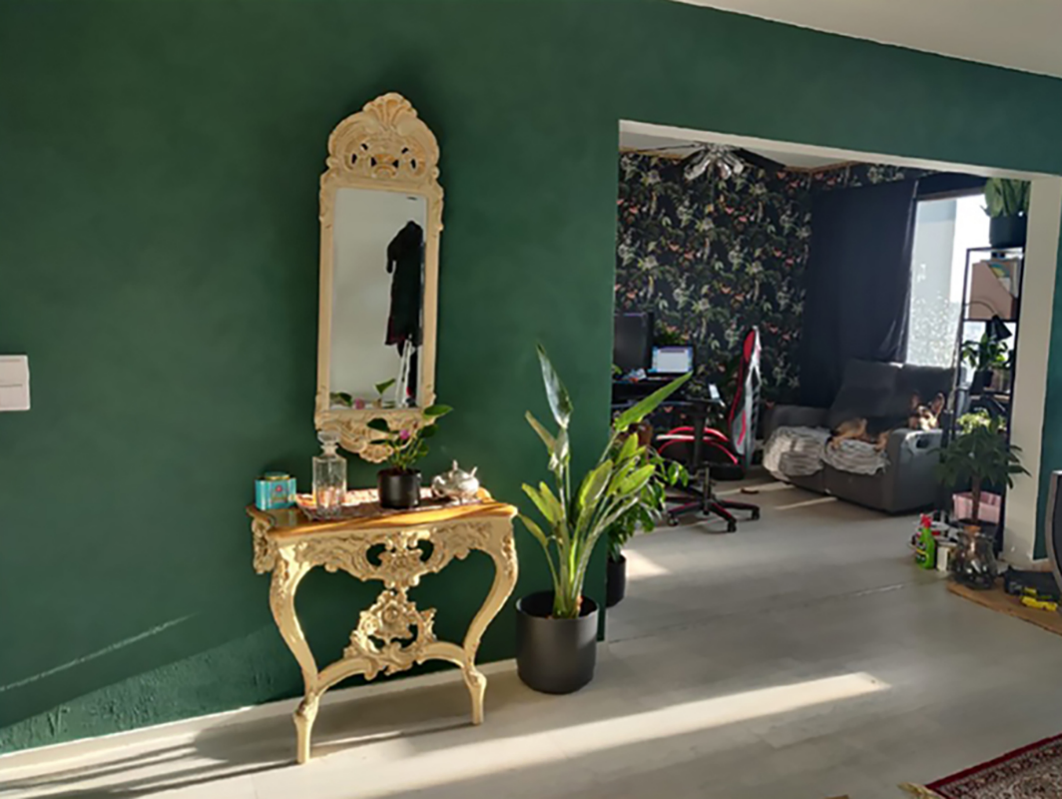
A home office in a separate nook connected to the living room. A dog rests on the sofa beside the workstation. Natural light is flowing from the window.

Regarding spaces, no participant mentioned having a separate room for office work at home. The most common places to perform office work at home were at the kitchen table, in an armchair or sofa in the living room, in-between spaces next to the household’s common spaces, or in the bedroom. Workstations were placed in a corner or “nook” in a room intended for other purposes, as one participant (participant 13; Figure 2) lucidly described it. Such working nooks were often narrow or cramped. On the other hand, these nooks seemed to be chosen with “...windows face [-ing] south, which [let] great light in from early to late afternoon” (participant 13) or “...quite magical views from my living room office space and [I’d] rather look at nature than miserable office walls” (participant 11).

An interesting quote we attribute to spaces describes a backpack as the perceived workplace:

*My backpack is my workplace–I have everything I need to do a good job in my backpack. Well, sometimes I need a bigger screen and a separate keyboard, but those usually exist in the places where I go to work. Working from the backpack is health-promoting and means that I am never out of anything*.

[Participant 3]

**Figure 3.**
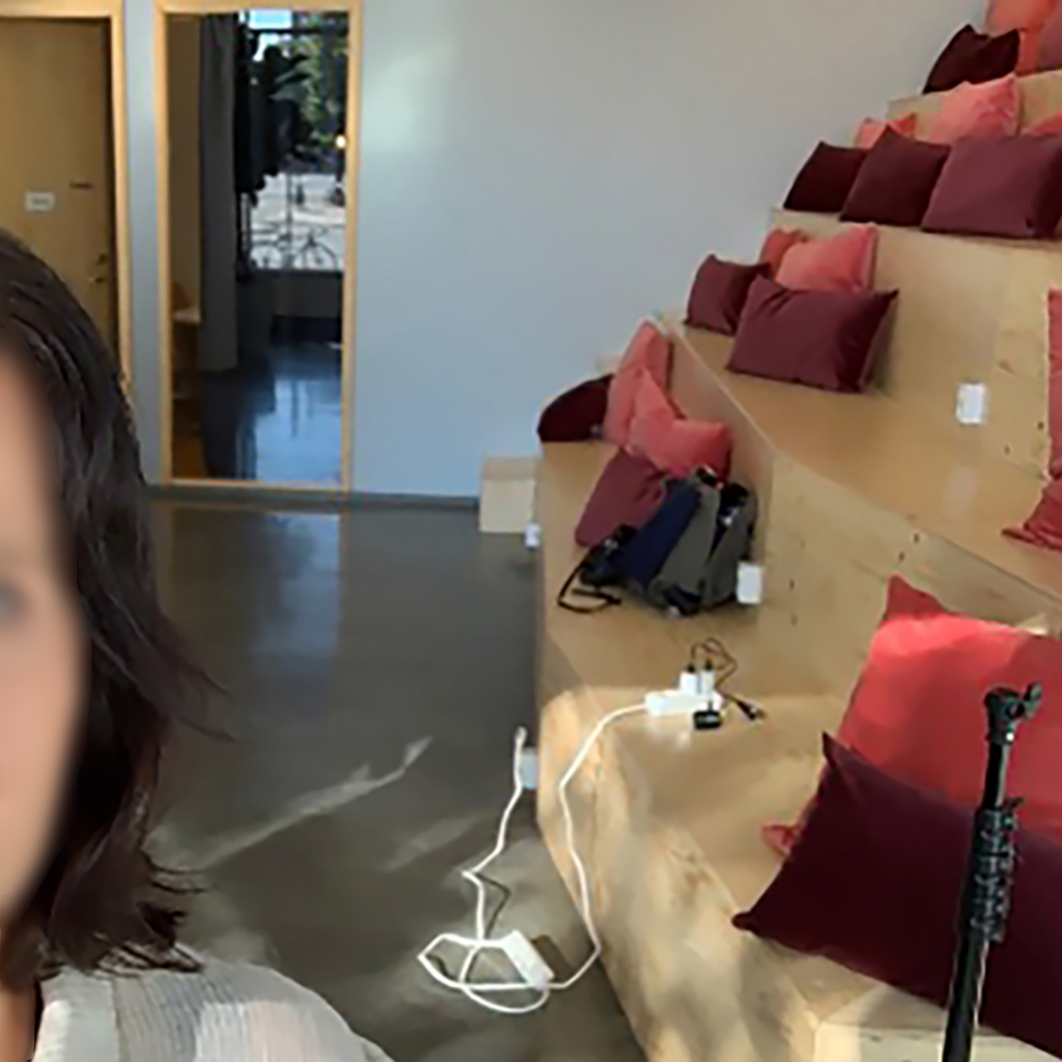
At the first level of this terraced structure, there is a backpack: “my workplace,” the participant explains. The photo was cropped, and the face was blurred to avoid showing the identity of the person who took the photo.

The quote illustrates a mobile working life and how one might perceive a space as small as a backpack as the workplace—a stretched space unbounded by time or place. This participant considered carrying “the office” with them as health-promoting (Participant 3; Figure 3).

Furthermore, participants named a balcony “with enough shadow for screen work” (Participant 2) and a cottage situated “close to nature” (Participant 3) as spaces where they performed office work at home, which are also enfolded in the aesthetic-sensuous and flexibility aspects.

**Figure 4.**
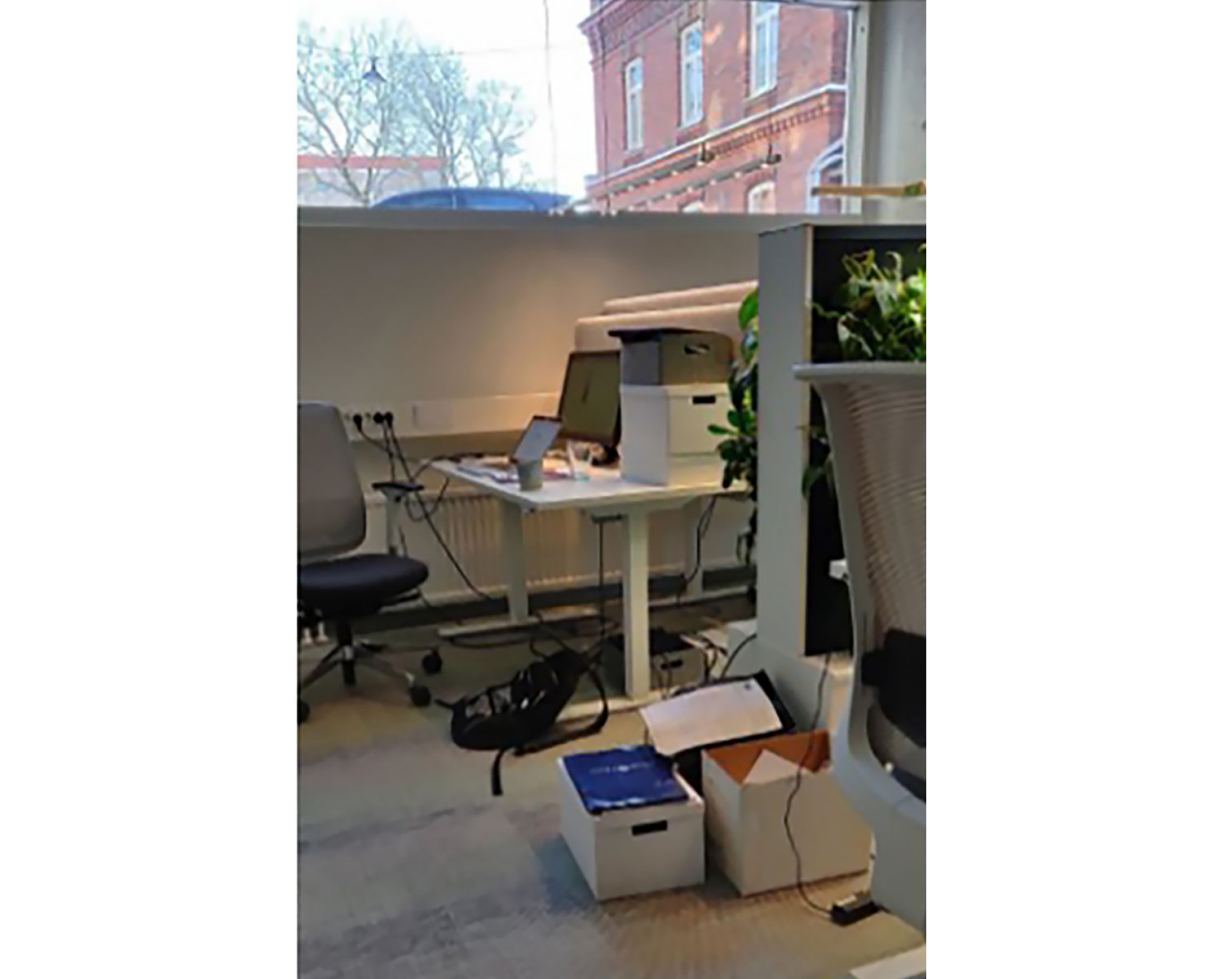
Left boxes and things were perceived as annoying and distracting.

**Figure 5.**
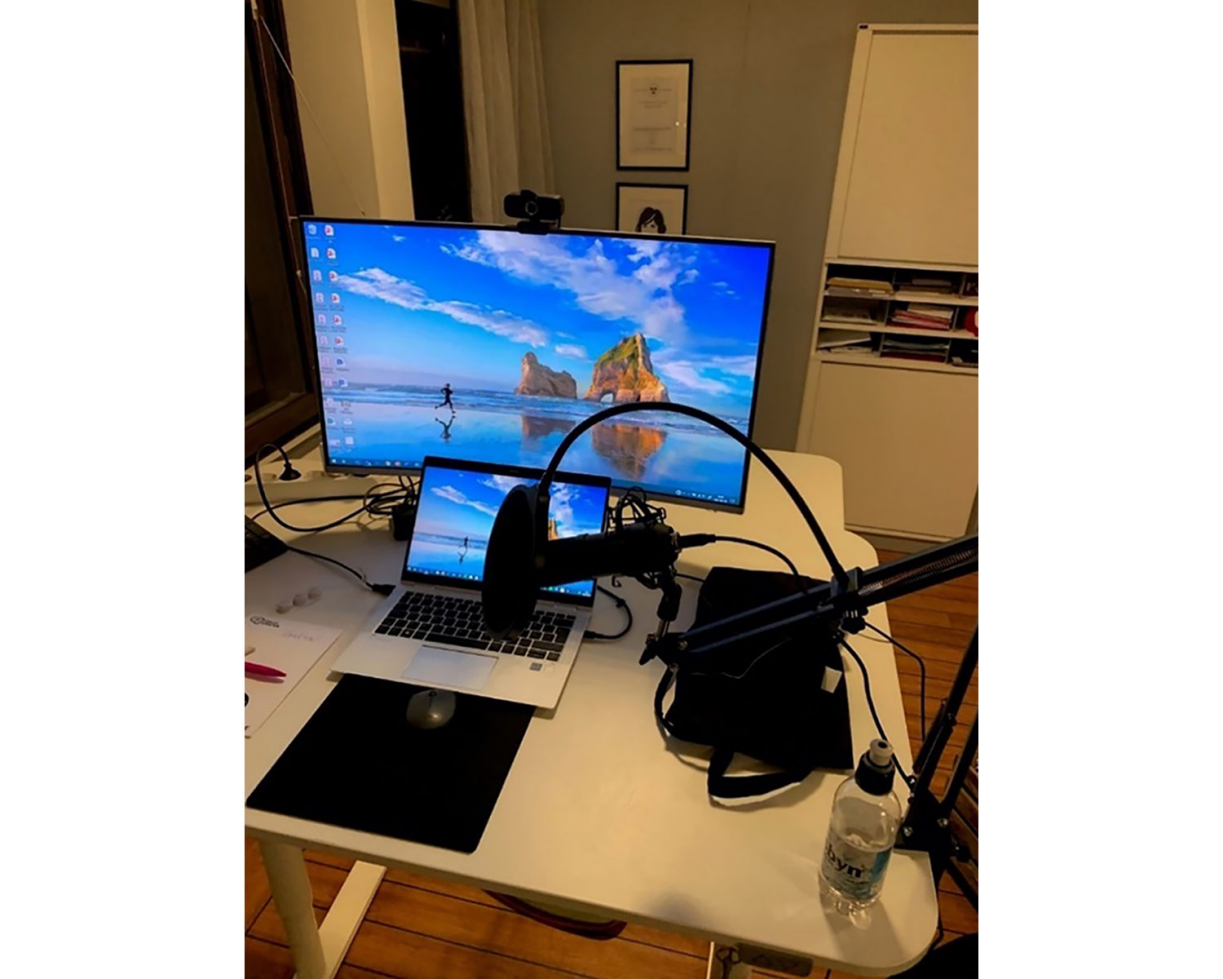
The desk became a video conferencing studio.

Another remark was that performing office work from home mainly happens in the middle of everything (eg, working on the sofa in a living room or at a kitchen table after other family members have arrived) and is therefore often perceived as messy. However, working at the office can also be messy or annoying, and demanding of one’s cognitive attention “when coworkers don’t clean up after themselves” (Participant 12; Figure 4). The “desktop also became a Teams studio [--] and the sound equipment had to be assembled and disassembled” to enable one to work with other tasks; this was not considered health-promoting (Participant 3; Figure 5). Keeping one’s workstation in order was easier if one had a separate workstation, whether at home or at the office.

#### Physical Ergonomics

Participants often considered physical ergonomics to be better at the office than at home, at least partly due to more appropriate equipment:

*At the office, I have a desk that can be raised and lowered which I appreciate. A chair that is easy to adjust. There is a balance board I can stand and swing on which provides much relief. [--] I have dual screens and I think that helps me a lot in my daily work. [--]*

[Participant 5]

**Figure 6.**
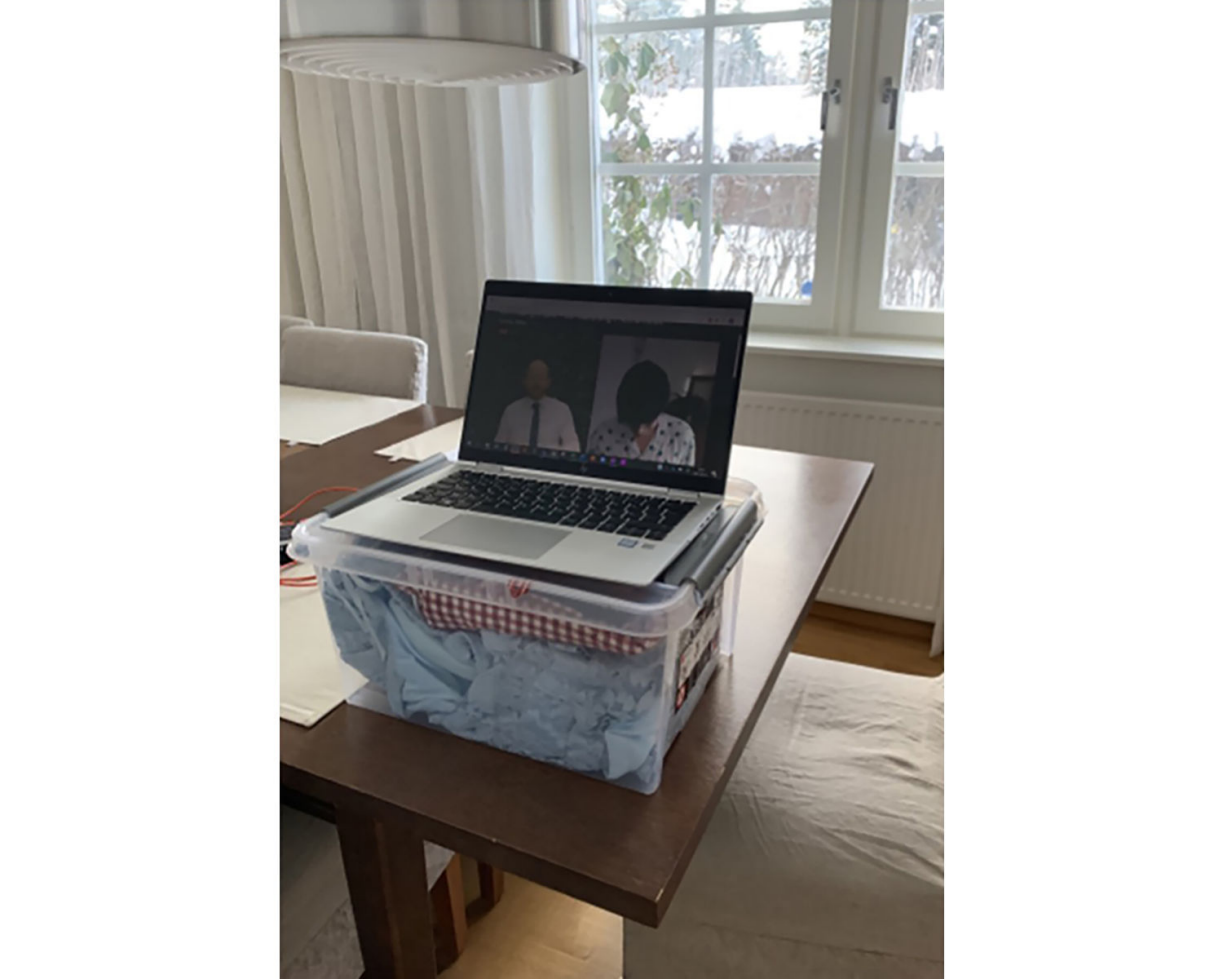
Simple solutions, such as storage boxes, were used to vary positions.

Ergonomic problems at home were solved by bringing in equipment from the office, buying equipment with one’s own money, or using simple solutions such as boxes, piles of books, and so on (Figure 6). Participants were aware of the importance of varying working positions, which was more difficult at home with less adapted equipment.

*I bought a “laptop desk” to keep the laptop higher on the table so I can stand. Now I can vary the position a bit, sometimes I sit, sometimes I stand. Nice and budget-friendly [--] no possibility of an electric table right now*.

[Participant 11]

**Figure 7.**
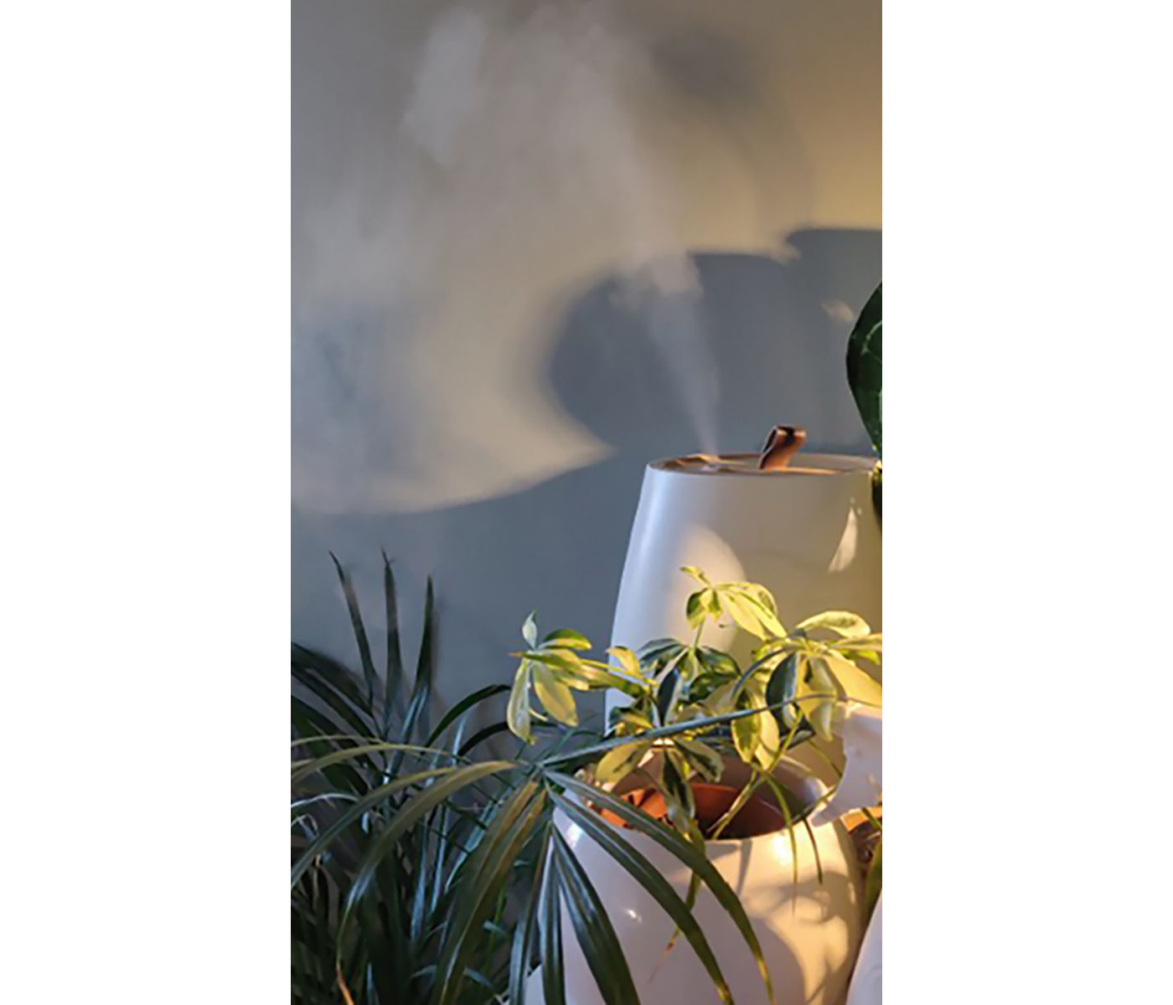
An air humidifier for improved air quality, in ensemble with indoor plants.

In contrast to artificial light, participants often highlighted daylight as positive, both at the office and at home. They also mentioned air quality as part of physical ergonomic requisites: “The air feels fresher and it’s easier to breathe, especially during winter, and my skin, eyes, and sinuses don’t get irritated as much” (Participant 14; Figure 7). In this example from a home office, an air humidifier was aesthetically in harmony with indoor plants.

#### Technical Aspects

**Figure 8.**
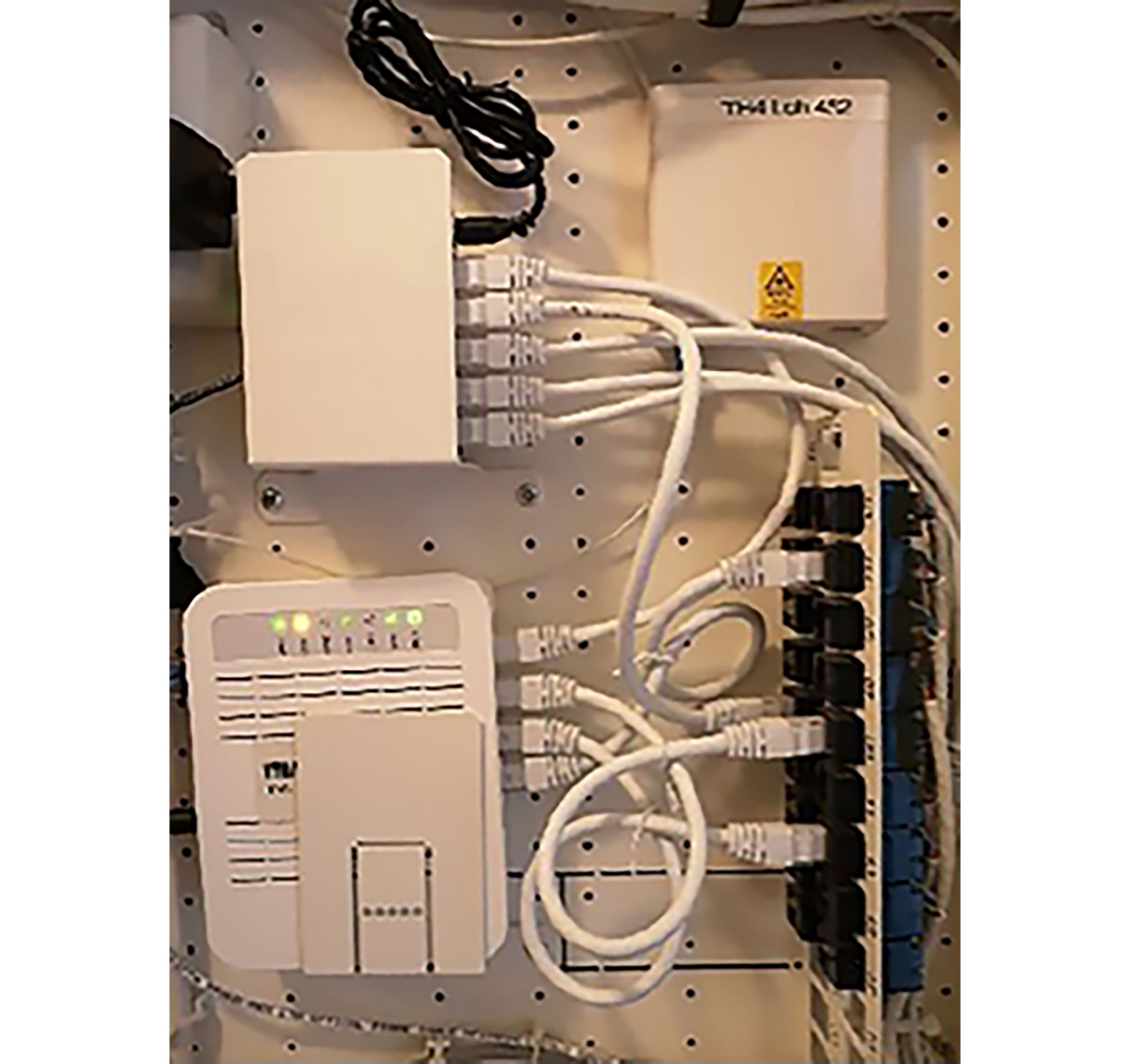
Stable digital connections are a prerequisite for presence and productivity.

Regarding technical aspects, poor technical solutions and a lack of equipment caused frustration during digital meetings, more so at home, but also at the office. On the other hand, participants named quick and stable digital connections as prerequisites for productivity (Figure 8).

Another concern was that one might be perceived as being absent from work if the internet momentarily goes down:

*Unreliable internet connections with several interruptions…creates frustration, especially when the sound disappears and the conversations are therefore [--] not as natural. There are also [--] concerns of this getting perceived as being offline even though you are working locally on the computer when the internet is down and [--] choppy*.

[Participant 17]

The quote above adds a layer of work-related norms to the frustration that insufficient internet connections create. Being present or in place is not enough; it is also important to be visible.

Technical aspects, such as a good internet connection and enough shade for screen work, were also deemed relevant when performing office work outdoors.

#### Aesthetic-Sensuous Aspect

The aesthetic-sensuous aspect was mirrored in several ways. A longing for nature was obvious both in the photos and in the comments, whether working outdoors or indoors at the office or at home:

*There are quite magical views from my living room office space, and [I’d] rather look at nature than miserable office walls*.

[Participant 11]

*[--] When I work from home, at our country house, I am close to nature. That’s health-supporting and even if I don’t always go outside, the surroundings have this calming effect on me*.

[Participant 3]

Many appreciated having more decorations and personal items at home, compared to the office, making their home office feel cozier and more aesthetically pleasing. It seemed more common to arrange for outdoor work or to bring natural elements inside when working from home.

Houseplants were observed and mentioned by participants as increasing coziness both at the office and at home. Interestingly, one participant even used the wording “aesthetically pleasing surrounding” (Participant 14) in relation to happiness and calmness:

*[--] Having an aesthetically pleasing surrounding makes me overall happier and calmer, and houseplants have a big part in it*.

[Participant 14]

Several workstations in home environments were placed in front of wallpaper with nature motifs. Another characteristic highlighted in relation to nature was that “unlimited daylight and inspiring [outdoor] environments” (Participant 2) were seen as a balancing factor between work and leisure. Participant 2 noted, “...it makes me feel like I’m getting a lot done, at the same time that it feels like a bit of a vacation.”

**Figure 9.**
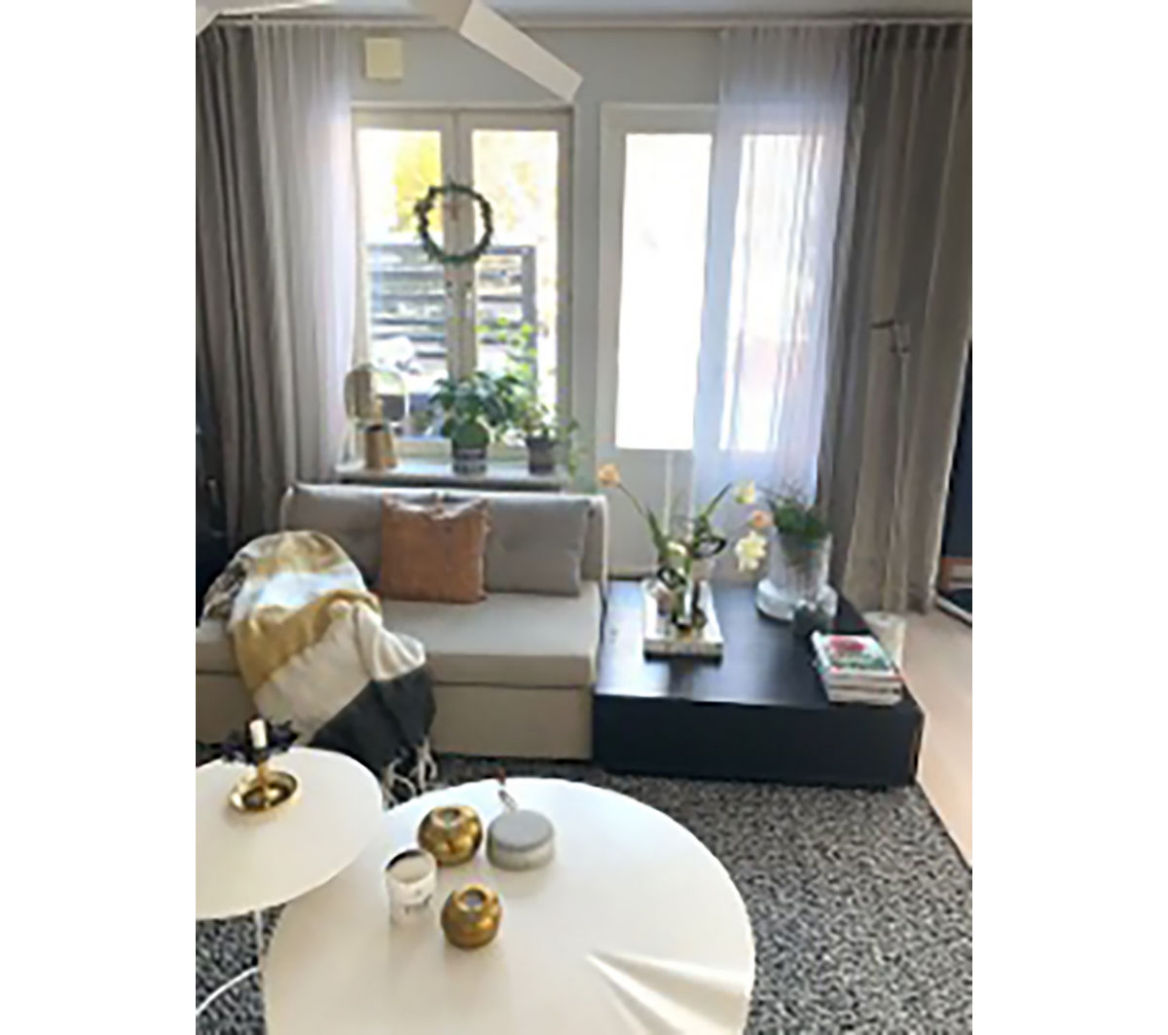
Flowing light, aesthetically pleasing things, a garden view, and coffee.

Figure 9 and the accompanying comment highlight several features that many participants considered supportive of health, such as views of nature and access to daylight, indoor decoration, and convenient proximity to important amenities such as the coffee machine.

*This is where I prefer to work when I'm inside [at home]. The light flows and I am happy to sit here. I can look out over the garden, and I am close to the coffee machine*.

[Participant 2]

Finally, a longing for colleagues was articulated when working from home, or in other words, the lack of social contact, creative exchange, and spontaneous interaction. This was only expressed in the comments (refer to quotes below), as taking photos of other people was not allowed:

*Loneliness and the lack of colleagues—even though it is nice that the noise level is low and that there are few disturbing moments, working from home becomes quite lonely during longer periods*.

[Participant 17]

*The social and creative exchange of a spontaneous meeting or coffee break is missing*.

[Participant 6]

#### Flexibility

Participants clearly appreciated flexibility and noted that they had the possibility “[--] to prioritize exercise during the daytime, doctor’s appointments, etc” (Participant 17). Even though working from home offered flexibility to arrange and distribute time more seamlessly, it also made it more difficult to create a balance between the two, as mirrored in the following quote:

*At the same time, there are often later days and more email checks during the evenings since the borders between private and professional life are blurred*.

[Participant 17]

Regarding work at the office, participants pinpointed flexibility in other dimensions; for example, having different collaborative environments or the possibility of bringing a dog to the formal office environment:

*We [are situated] in an office hotel and there you can bring your dog to work [--]. That’s health-supporting and [--] allows me to relax since I don't have to ask someone else to look after him, and my colleagues appreciate him*.

[Participant 3]

#### Focus

Regarding focus when working from home, participants said that silence and fewer work-related distractions were helpful. On the other hand, activities unrelated to work, such as domestic logistics with responsibility for children, messiness at home, and easier access to snacks, caused distractions. Several participants reported difficulties in maintaining boundaries between work and private life. Furthermore, frustration over poor internet connections impaired focus:

*A large, stylish desk by the window, the silence that enables concentration and efficient work. [--] Disadvantages: before starting work, one must clear the house and it’s hard to get started when you see so much that needs to be done. It’s also difficult to stop working in the evening. Sometimes, we go on without taking a break and forget to have lunch!*

[Participant 9]

*[--] It’s nice that the noise level is low and that there are only a few distractions. But it’s much more difficult to balance private and professional life. However, it gives more flexibility [--] to prioritize exercise during the daytime, doctor’s appointments, etc. At the same time, there are often later days and more email checks during the evenings since the borders between private and professional life are blurred*.

[Participant 17]

How much focus was impacted depended on whether the participant was at home alone or in the company of other family members who were not working:

*This [kitchen] is quite a challenging place to focus sometimes because there are also other “home officers” [others who also work from home] here so they keep coming and talking and eating and getting some coffee all the time*.

[Participant 11]

**Figure 10.**
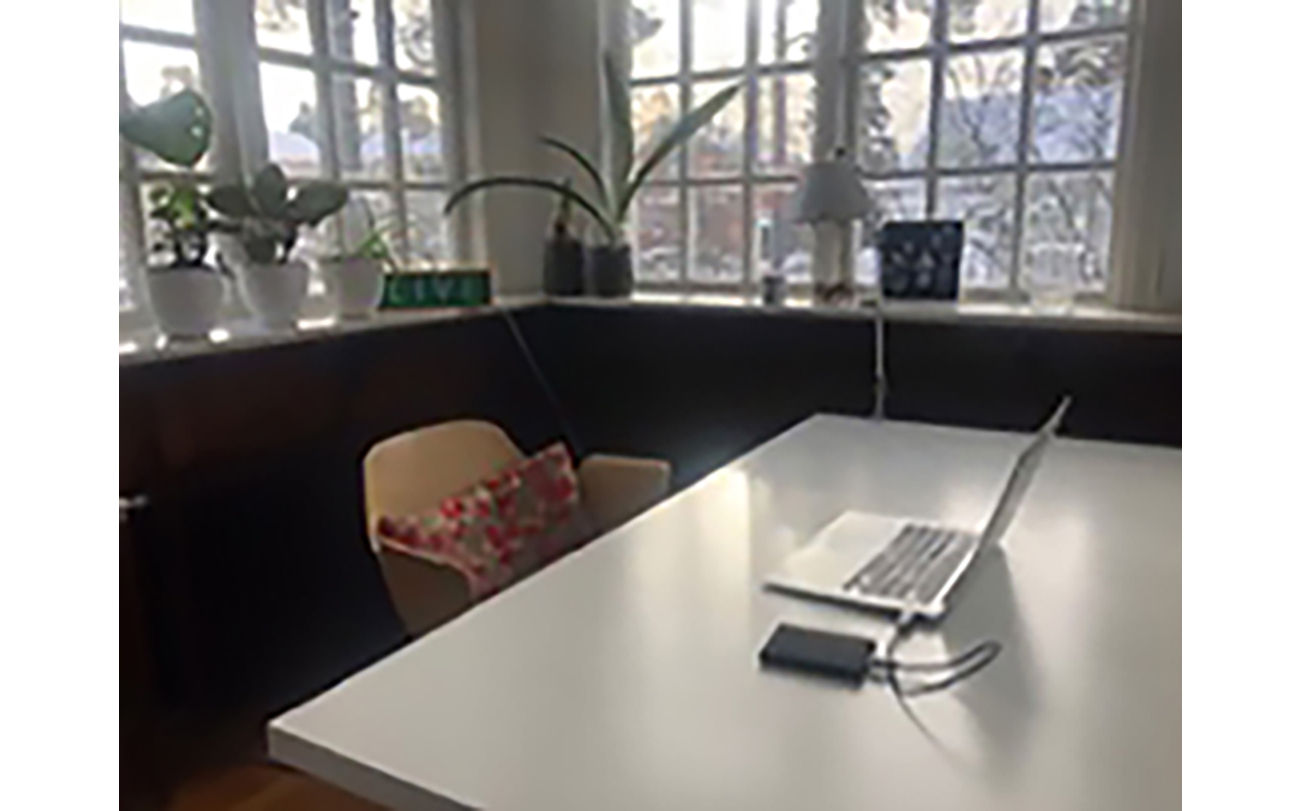
The workday, from home, starts with a clean desk and silence.

**Figure 11.**
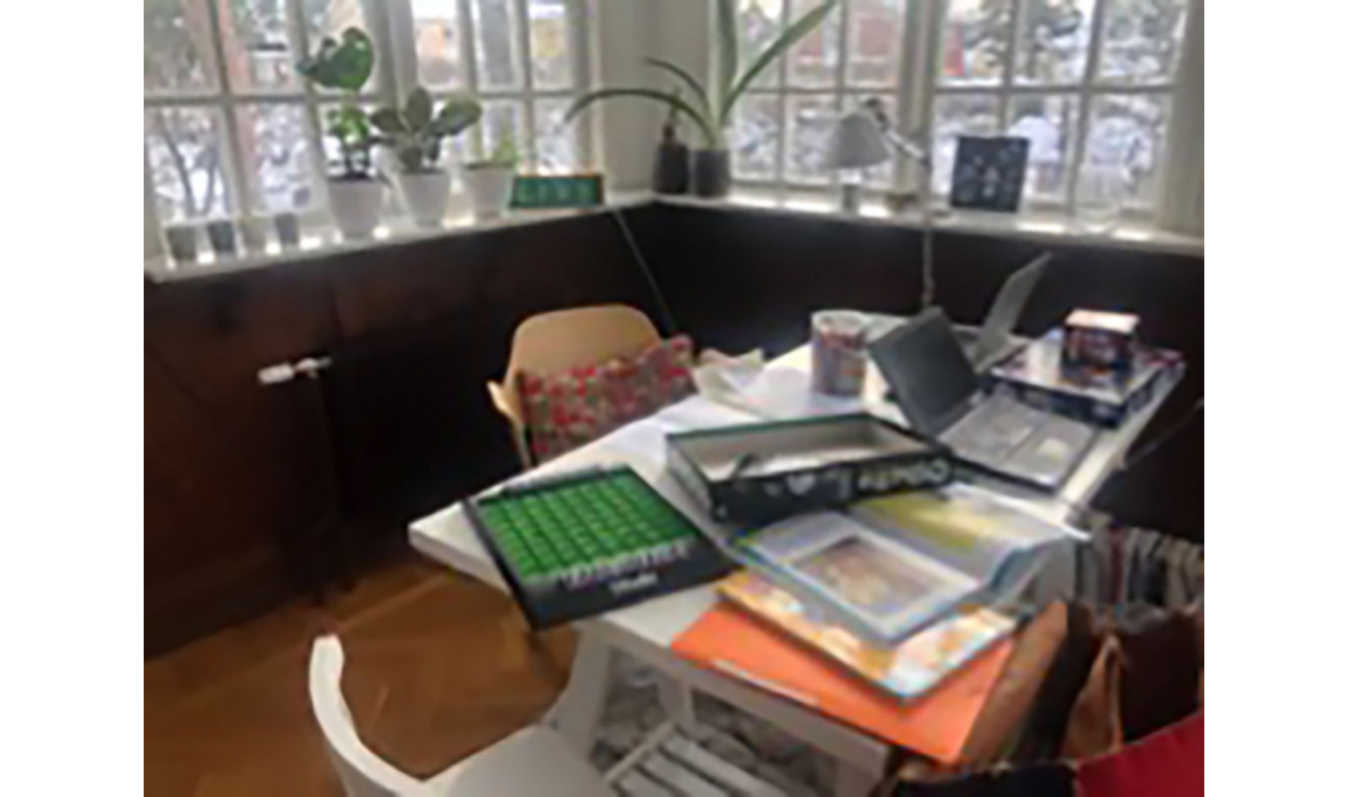
At 4 PM, the desk gets crowded with other activities.

Figures 10 and 11, with their respective comments, highlight the domestic transformation associated with working from home. The workday begins alone at the almost empty “...big, neat desk by the window. The silence allows for concentration and efficient work” (Participant 9; Figure 10). Around 4 PM, the scenery begins to change when other family members arrive, starting their activities and disrupting focus.

*Advantages: At 4 pm. the children arrive home. The working day is over! Unpleasant: the office becomes busy with other activities. Drawing, playing, doing homework, [--] plus I don't have a printer*.

[Participant 9]

There is an abrupt stop to the workday and no time to mentally transition from work mode to parenting mode or to summarize the day’s work, as one often does when commuting (Participant 9; Figure 11).

#### Breaks-Recovery

Concerning breaks and recovery*,* many participants working from home referred to “active breaks,” including physical activity, meeting friends, or listening to a podcast. This indicates that it is easier to take active breaks when working from home since it “saves time [and] no transportation [is] needed. One can easily take a break and run or do yoga” (Participant 9). However, active breaks were also possible when working in the office, such as by using exercise equipment or going for a lunch walk. Participants expressed that attractive views could act as a trigger that “inspires one to go out for a lunchtime walk” (Participant 1) but also gives one “a chance to have mini breaks while working simply by letting...[one’s] eyes wander outside” (Participant 14). Another example of a break not associated with physical activity is: “A calm coffee room improves well-being at the workplace as you can get a break and don’t have to look at others working” (Participant 12). Still, the following quote highlights recovery even without taking breaks:

*To the left, you see a glass wall facing our meeting room. We have access to ‘quiet’ rooms. We have several [--] units that provide optimized humidity and neutralized air flow during the working day. We also have extra daylight luminaires that support the individual’s circadian rhythm. To optimize focus, performance, and recovery*.

[Participant 10]

The quote above is from an office and describes technically advanced equipment intended to simultaneously support recovery and work performance. However, the atmosphere at home can be perceived as work-supporting, as reflected in the three quotes below:

*A safe environment with a cozy atmosphere, perfect for reading and some recovery*.

[Participant 15]

*Relaxing and warming with a fireplace. Creates a nice atmosphere to work in*.

[Participant 15]

*In the home office I also have an assistant [my dog] who is always ready to help me to remember the little breaks and keeps me focused in positive vibes*.

[Participant 11]

The 3 latter quotes, as well as previously mentioned comments on views, also exemplify the interconnection between breaks-recovery and aesthetic-sensuous aspects.

#### Physical Activity

**Figure 12.**
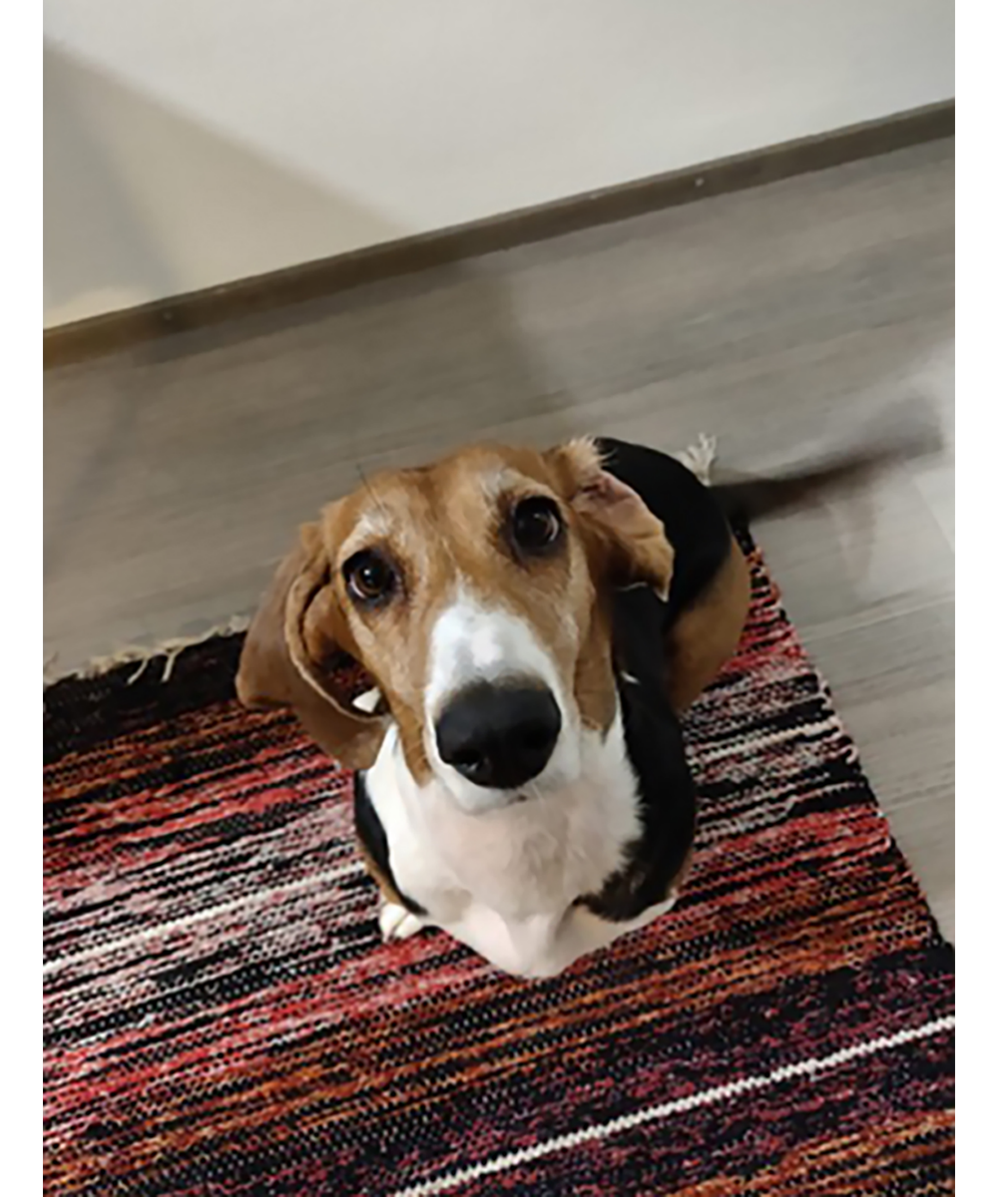
This dog is alerting the need for small breaks for movement, but it also brings good vibes and company during the workday.

Another aspect was physical activity*,* linking back to breaks and recovery. Physical activities mentioned by participants working from home included walking, running, doing yoga, gardening, and taking short swims. Closeness to nature, beautiful surroundings, and having a dog were mentioned as motivating factors. “My dog keeps watch on our break times and never fails to inform me about sitting for too long” (Participant 13; Figure 12). Also, sporadically, walk-and-talk meetings were mentioned.

When working in the office, physical activity seemed to become a planned part of the working day, for example, in the form of active transportation (eg, walking or biking) to or from work. Commuting is time-consuming in comparison to working from home, but “I usually get off a few stations earlier to accumulate 10,000 steps. It feels like these walks are becoming part of getting to work and home.”

[Participant 5]

#### Eating Habits

Finally, eating habits were another aspect identified in the written comments, although they were not as common as physical activity. These comments were quite varied; some participants stated that they eat better at home, while others eat better at the office. Maintaining healthy eating habits may be challenged by easy access to unhealthy snacks or a lack of healthy lunch alternatives, as reflected in the following comments:

*I think I eat better at home than at work [the office]. We are good about buying healthy food. I'm not a person who likes to snack, so for me, that’s not a problem*.

[Participant 5]

*[--] Also, the close vicinity of the fridge [at home] makes snacking while working too easy*.

[Participant 13]

*[--] Food is lacking [at the office] sometimes, when I forget to bring my lunch box or a snack [--] something less good might slip down in the afternoon. We have a good kitchenette with a microwave so there is no blame*.

[Participant 5]

One can also forget to eat lunch when working from home (Participant 9). A comment categorized as breaks-recovery, referring to “a calm coffee room” that allows for breaks without seeing colleagues working (Participant 12), can also be interpreted as a health-promoting quality related to eating habits.

### Visual Framework Derived From the Identified Aspects

**Figure 13.**
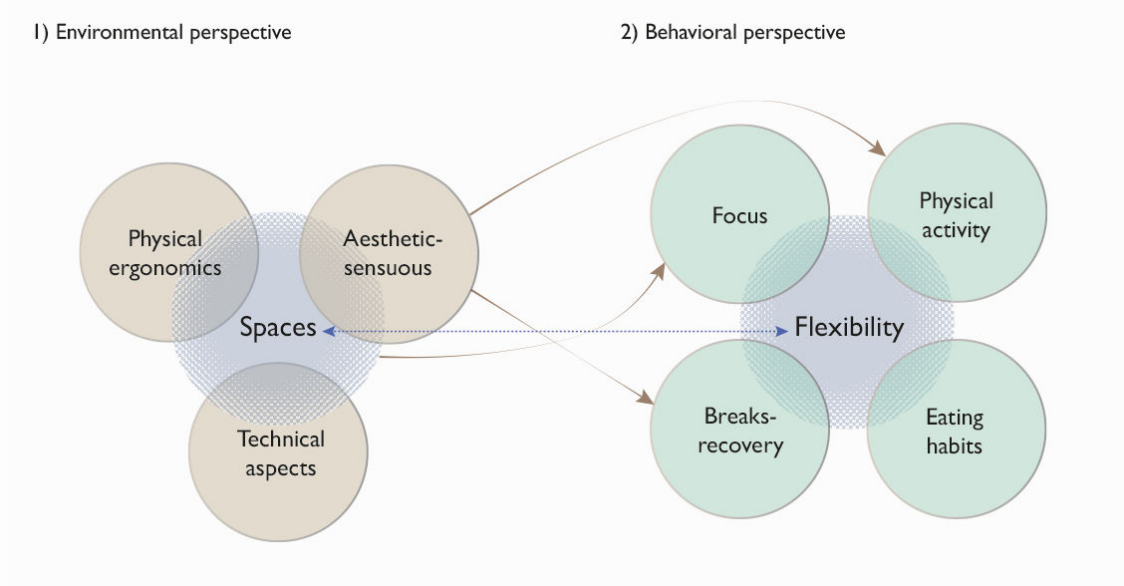
Visual framework derived from the nine identified aspects considered to support or hinder health in places where office work is performed, clustered into two groups: environmental perspective and behavioral perspective. Spaces bind together the three aspects of physical ergonomics, aesthetic-sensuous, and technical, while flexibility binds together the other four aspects: focus, physical activity, breaks-recovery, and eating habits. The dashed 2-way arrow (blue) indicates that spaces and flexibility bridge the environmental and behavioral perspectives with their related aspects. The solid arrows (brown) indicate connections between specific aspects in the 2 perspectives.

The visual framework (Figure 13) summarizes how the nine aspects were organized into two clusters: an environmental perspective (spaces, physical ergonomics, technical, and aesthetic-sensuous) and a behavioral perspective (flexibility, focus, breaks-recovery, physical activity, and eating habits).

Regarding the environmental perspective, spaces are the connecting aspect within this group, as we all work in constructed environments, traditionally in offices shaped by employers. The aesthetic-sensuous aspect concerns qualities in one’s surroundings and in objects that “pull [one] out of the everyday for just a moment” via their beauty [36]. It can be how light is flowing, a view, a plant, or an aesthetically pleasing object that provides the feeling of calmness or happiness, and in turn allows for microbreaks. There are connections between the aesthetic-sensuous aspect (within the environmental perspective) and the aspects of physical activity and breaks-recovery (within the behavioral perspective). This is because breaks and recovery may also involve experiences of joy and calmness when meeting colleagues, as well as creative ways of relaxing while simultaneously gaining new ideas and impulses. In this sense, recovery can occur together with colleagues. This is why the aspect is not referred to simply as aesthetic, but as aesthetic-sensuous: it captures the dynamic sensitivity present in human interactions that operate on a more sensuous level.

Regarding the behavioral perspective, we interpreted flexibility as the connecting aspect within this group as follows: the constructed environments where we work are populated by individuals with different needs and preconditions. Flexibility can be seen as an enabler for individuals to adapt their life puzzles based on specific needs regarding place, space, and time in private and professional life. At the same time, it makes it more difficult to create a balance between the two, eventually causing distractions and affecting focus. This duality was expressed by several participants in relation to working from home. Regarding working at the office, flexibility was described differently, for example, in the form of being able to bring a dog, having access to different collaborative environments, or interpreting one’s backpack as a flexible workplace.

Spaces and flexibility are connecting aspects that bridge the environmental and behavioral perspectives with their related aspects (Figure 13). This highlights that flexibility is not only related to the behavioral perspective but also to the environmental perspective because it enables the individual to use work environments in relation to different needs. Furthermore, organizational flexibility as such is part of the bigger interplay of values related to the environmental perspective. Spaces must be designed and organized with this interplay in mind, that is, with flexibility allowing for personalized solutions.

The framework also includes connections between specific aspects in the 2 clusters (Figure 13); aesthetic-sensuous aspects, such as beautiful views, attracted active breaks with physical activity as well as breaks-recovery without any physical activity or even while working. Spaces originally meant for other purposes were messy or populated by other people, causing distractions and affecting focus.

The company representatives who attended the last annual SOFCO workshop confirmed the value of the visual framework, as it illustrates how various environmental and behavioral aspects are interconnected. They considered it useful for understanding the whole picture and the interrelationships between these aspects.

### The Battery of Multiple-Choice Questions About Office Work

Regarding the second objective, the battery of multiple-choice questions about office work is presented in Multimedia Appendix 2. The battery includes nine questions that are all presented twice to cover two time periods: “before the COVID-19 pandemic” and “at present” (ie, postpandemic period). The questions cover the following four settings where office work might be conducted: (1) work in an office environment (the room or premises the employer offers), with 4 questions about working time (days per week) in the office, office type (eg, cell office, open-plan office of different sizes, activity-based flex office, and so on), access to a room for specific tasks that may require silence, and experience of whether the office environment allows one to do a good job. The response alternatives to the question on working time in an office environment were modified to represent the number of days per week instead of different proportions of total working time, as in the 2019 Swedish Work Environment Survey [30]. In addition, the option “Don’t know/Don’t wish to answer” was added to all four questions. (2) Office work performed at home, with three questions about working time (days per week) at home, access to a room for specific tasks that may require silence, and perception of whether working from home allows one to do a good job. In addition, there is (3) one question about the frequency of performing office work in cafes, restaurants, hotel lobbies, or similar public places, and (4) a similar question related to office work performed outdoors.

## Discussion

### Summary of Main Findings

In line with objective 1, nine significant aspects stood out in the material: aesthetic-sensuous, breaks-recovery, eating habits, flexibility, focus, physical activity, physical ergonomics, spaces, and technical. These aspects were synthesized into a visual framework (Figure 13) that clusters them into environmental and behavioral perspectives and depicts their interconnections, with spaces and flexibility bridging the 2 perspectives.

In line with objective 2, the study resulted in a battery of multiple-choice questions for quantitative studies that extends office-work questions beyond conventional office environments to include office work performed at home, in public places (eg, cafes or hotel lobbies), and outdoors. The questions are presented for 2 time periods (“before the COVID-19 pandemic” and “at present”).

### Present Findings in Relation to Previous Research

Regarding objective 1, our visual framework aligns well with recent Swedish interview findings on remote work during the COVID-19 pandemic [16], where 5 overarching themes captured both opportunities and challenges. Several of these themes map directly onto our aspects—particularly spaces, physical ergonomics, and technical prerequisites, as well as the duality of focus (efficacy) and aesthetic-sensuous (social isolation) and the links between flexibility and boundary management. Similarly, a scoping review on remote work and health among white-collar workers during the COVID-19 pandemic [11] identified several determinants of well-being—feelings of isolation and loneliness, and home office adaptability, including ergonomic factors and technical support—that align with our aspects. The scoping review [11] corroborates our findings that performing office work at home can have both supportive and hindering impacts on health, depending on other contextual factors.

The environmental perspective (spaces, physical ergonomics, technical, and aesthetic-sensuous aspects) of our visual framework also resonates with findings from 2 studies that complement each other. In a quantitative study in the United States, workstation furniture was rated higher in the office, while comfort characteristics such as temperature, air quality, noise, natural light, outdoor views and access, adequate space or privacy, and overall aesthetics were rated higher at home [12]. Yet, attributes such as plants, daylight, outdoor views, warm colors, and lower occupancy can be important for psychological and cognitive responses also in office environments, according to an experimental study in the Netherlands [4]. Furthermore, in a very recent US study, participants rated decorated (a rug, faux plants, artwork, and a nicer desk and chair) university rooms as more pleasant and comfortable and reported greater interest in working in these rooms compared to nondecorated rooms [37].

The respondents of this study mentioned that while the ergonomics were often poorer at home than in the office, the home environment was cozier and more atmospheric. The same argument was used by managers in charge of office premises in a study on organizational strategies for office working life after COVID-19, who reported that employees would choose to work from home despite poorer workstation ergonomics [38].

Regarding longing for colleagues, previous research suggests that regular social support from colleagues and employers when working from home during the pandemic may mitigate stress and promote well-being [11], while extensive use of digital tools may lead to techno-fatigue or other types of technostress [39], which relates to our technical and focus aspects. On the other hand, working from home may increase connectedness with family and friends [12]. A well-balanced and tailored hybrid work approach—with flexibility in space, time, and digital tools [18], including optimized design of the different environments where office work is performed [12]—may offer a partial solution. Whether and to what degree flexibility and autonomy are allowed by the spatial design and use of space depend on the organizational culture and management style [40]. Consequently, office design has become a strategic management tool in contemporary office working life [41], including hybrid work. Further research is needed to find evidence-based solutions for employees who only or mostly work from home, in particular [11].

Considering the behavioral perspective, the flexibility aspect corresponds to prior descriptions of increased autonomy and boundary control in telework, as well as challenges in maintaining work-life boundaries [11,16]. With respect to physical activity, individual differences in needs, prerequisites, and barriers have been highlighted by previous studies, including during the COVID-19 pandemic [42]. While substantial evidence exists on physical activity in office contexts [43], remote and hybrid work may imply different conditions for movement and recovery, underscoring the need to consider home-based work settings in recommendations and interventions [13,42,44]. For eating habits, earlier work suggests that factors in office-based work can negatively influence eating [10], and reviews indicate that nutritional interventions among office workers remain limited [45].

Our findings also resonate with the salutogenic workplace environment framework by Roskams and Haynes [3], which conceptualizes environmental resources as supporting an employee’s sense of coherence (comprehensibility, manageability, and meaningfulness). In particular, the aesthetic-sensuous, breaks-recovery, and physical activity aspects are consistent with the idea that manageability of everyday work can be supported by bringing nature or mimicking nature into the workplace through design that supports social cohesion, including spaces that encourage breaks and by promoting physical activity [3]. Furthermore, a way to promote meaningfulness is to allow (or encourage) employees to decorate their workspace with personal items or photographs [3].

Several aspects identified in this study relate to factors that have been discussed in relation to self-reported productivity of white-collar workers during the COVID-19 pandemic, both when working at the office and at home [11,12,46]. The pathway from such factors (eg, social isolation, distractions, lack of regular office space or technical support, workstation furniture, and overall aesthetics [11,12]) to productivity is probably complex and may partly be mediated by the health and well-being of employees. While we cannot make any conclusions about productivity, this previous research [11,12,46] may suggest that promoting healthy lifestyles at work could both support employees and benefit employers, the health care system, and society at large.

Altogether, for objective 1, our findings are consistent with prior research demonstrating that multiple workplace attributes matter simultaneously, and that interactions between attributes can be important for outcomes such as comfort and connectedness [14]. Extending this literature, our visual framework summarizes not only environmental aspects but also behavioral aspects relevant to health during office work and illustrates interactions both within and between the environmental and behavioral perspectives. Another novelty of this study is that, while previous research suggests that aspects of well-being may differ between home and office settings [12,13], our framework is designed to be applicable to office work performed across settings. Our findings indicate that supportive and hindering factors exist in both office and home environments, although their importance may differ by context. While we believe that the framework can be used as a structure for tailoring interventions in both office and home environments—including hybrid work—we suggest that this duality and the applicability of our framework to multiple work environments should be further evaluated.

Concerning practice-oriented implications of our results, the 9 aspects overlap with established building standards that include health-related components for work (eg, WELL Building Standard [47] and Fitwel Certification [48]). Our visual framework can complement such standards by making behavioral aspects and interactions between factors more explicit.

Regarding objective 2, the rapid changes in where office work was performed during the COVID-19 pandemic were mirrored in our photovoice material and underscored the need for survey questions that capture office work beyond conventional offices. The questions were developed by adapting items from the 2019 Swedish Work Environment Survey [30] to cover additional work settings beyond the office. Since then, the Swedish Work Environment Authority has updated its survey (2021) by differentiating desk work at home from desk work in office environments outside the home, such as coworking spaces [49], and other large surveys (eg, The Swedish Longitudinal Occupational Survey of Health [50]) have added questions about working at home vs in an office during and after the pandemic. However, these instruments typically do not include specific questions about office work performed outdoors or in public places, which are covered by the new questions presented in this study.

### Methodological Considerations and Future Research

At the time of the study, during the COVID-19 pandemic, prerequisites for working from home were often debated in the media; it can therefore be assumed that awareness of conditions for physical and psychological health and well-being had increased among office workers in general. On the other hand, we recruited participants from companies involved in the SOFCO project, that is, companies within the office sector, through designated contact persons. The participants had relatively high socioeconomic positions, which are typically associated with better health literacy. Higher health literacy, in turn, is associated with healthier behaviors and may mediate the association between socioeconomic factors and health behaviors [51].

Furthermore, the experience of working from home may differ substantially across age groups and between men and women [11,46]. In this study, the majority of the participants were women, and we lacked information on the age distribution of the sample. While our recruitment approach supported feasibility and access to relevant experiences, it may also have introduced self-selection bias and limited the transferability of the findings to other contexts. Future research should examine whether the proposed framework applies to more general and heterogeneous samples of office workers.

The pandemic also affected our data collection. Due to restrictions on physical meetings and limited adaptation to digital interaction, it was not possible to conduct interactive in-person sessions with participants. Consequently, we designed the photovoice study to allow for the digital collection of both photographs and written comments. We also obtained input from company representatives during 2 iterations of the SOFCO project’s annual workshops.

As the data collection was carried out shortly after the onset of the pandemic, the transferability of the findings to today’s voluntary hybrid working arrangements warrants discussion. Difficulties in adapting to digital interaction have likely decreased, ergonomic solutions may have improved, and experiences of loneliness associated with working from home may be less pronounced in hybrid work settings, partly due to advances in technical solutions. At the same time, our findings point to more general mechanisms—namely, what is perceived to support or hinder health during office work—rather than merely reflecting the effects of an abrupt transition to working from home. Our framework can therefore serve as an analytical tool for understanding health-related aspects of both office-based and home-based work environments, even as work organization continues to evolve.

Finally, methodological considerations not related to the pandemic include the fact that the reason 2 organizations did not provide data remains unknown. In addition, due to an external deadline for submitting new questions to the LifeGene project, we were unable to extend the recruitment or data collection periods.

### Conclusions

This study extends the notion that office environments play a crucial role in supporting employee health by suggesting that the same holds true for home offices. The 9 aspects identified in this study can either support or hinder health, depending on the environment in which office work is performed and employees’ living conditions. A key contribution of this study is the synthesis of the aspects into a visual framework that integrates an environmental perspective with a behavioral perspective and depicts interactions within and between them.

Our findings suggest that health promotion in the workplace must be tailored to accommodate multiple environments and individual living conditions where office work is performed. We recommend that health-promoting aspects are considered in all settings where office work takes place—whether in traditional offices, at home, or in hybrid arrangements that include other environments such as outdoor spaces, cafes, hotel lobbies, or coworking spaces—to a greater extent than before.

For research on lifestyles and health among office workers, it is central that survey questions about office work capture the diverse environments in which contemporary office work is performed. The battery of questions developed in this study, now integrated into the Swedish LifeGene study, extends existing question batteries by adding specific questions about office work outdoors or in public places, and 2 separate time perspectives. Thus, it offers one approach to measure office work across settings and time and may facilitate future quantitative studies on office work.

Our visual framework adds to the understanding of aspects promoting or hindering health while performing office work. Together, this framework and the developed survey questions capture the variation in modern office work arrangements, and they may thereby contribute to advancing sustainable, health-promoting offices of the future.

## Supplementary material

10.2196/90712 Multimedia Appendix 1 Results from the initial formal analysis, the first Concepts for the Sustainable Office of the Future workshop, and the revisiting of the photos together with the respective written comments.

10.2196/90712 Multimedia Appendix 2 Battery of multiple-choice questions about office work.

## Abbreviations

NWW: new ways of working
SOFCO: Concepts for the Sustainable Office of the Future
