# Supplementary material for: Framework for Health-Promoting Environments for Office Workers: Photovoice Study
Source: JMIR Form Res. 2026 Jun 29;10:e90712. doi: 10.2196/90712 (PMC13313410; doi:10.2196/90712)
Supplement: Multimedia Appendix 1 [file formative-v10-e90712-s001.docx]

This is a Multimedia Appendix to a full manuscript published in JMIR Formative Research. For full copyright and citation information see doi:[**10.2196/90712**](https://doi.org/10.2196/90712)

**Results from the initial formal analysis, the first SOFCO workshop, and the revisiting of the photos together with the respective written comments**

**The formal photo analysis** indicated that a majority of the photos represented the following: somewhere to sit down (different types of sitting furniture) at home; surfaces to place a computer, foremost a laptop (different types of tables, sideboards, chests of drawers) at home: other equipment for digital work (such as cables and routers); and elements that separate rooms (sound absorbers) in the offices. Additionally, cups, mugs, and water glasses were present both at home and in the office environments.

Photos taken in the home environment showed far more personal items but also plants and equipment for domestic work (e.g., washing machines). Storage in the form of bags and boxes was common, while only a few bookshelves or equivalent were detected in the photos. The boxes were also used to create better ergonomic conditions when working from home.

Windows were a dominant theme both in the offices and home environments. Especially in home settings, workstations were frequently placed close to windows with views showing gardens, trees, other houses, and the sky. Outdoor environments captured through the windows frequently pictured urban green spaces or gardens.

More sporadically, the photos showed balance boards, pets (dogs), rings for gymnastics at the office, a fireplace, an air humidifier at home, and a summer place in the countryside, for example.

In summary, the things shown in the photos presented and represented nature, light, ergonomics, prerequisites for work, physical activity, cramped spaces, order and disorder, and the mixing of work and private life at home. Altogether, this points towards the importance of conditions for carrying out one’s work in a satisfactory and sustainable way; that is, prerequisites for physical and psychological health.

In summary, break-out room discussions in the SOFCO workshop 1 include the following: In the home office environment, it is challenging to distinguish between work and leisure time. However, this situation also brings both opportunities and challenges due to the new flexibility of working from home. This duality is reflected not only in the work-life balance but also in aspects of the home office environment, such as its beauty (including light, color, and views) and its impracticalities (for example, ergonomical and technical issues).

**Adding written comments to the results of the formal photo analysis** showed that the comments usually consisted of a description and explanation of components. A dominating number of participant comments started with a description of the photo contents, followed by an explanation of what they regarded as supporting or hindering health at work. In general, the written comments showed the following: At home, one often had poorer ergonomics than at the office, both physically and regarding work equipment. At the same time, it was more cozy and atmospheric at home. Poor ergonomics were solved differently among participants, either in simple ways such as with piles of books or a laundry basket under the laptop, or in more advanced ways, such as with ergonomic standing mats or stand-up desk workstations. Furthermore, it was sometimes very cramped at home and one may work and sleep or eat in the same space. In such cases, equipment had to be assembled and disassembled daily. Chairs at home were often described as uncomfortable; on the other hand, the possibility of varying sitting positions using sofas or armchairs was described as positive. The importance of having a working Wi-Fi and mobile phone at home or when working outdoors was mentioned several times as a prerequisite for health-promoting office work.

There were varying opinions on whether one eats better at home or in the office and why; however, coffee was frequently articulated and visualized as a necessity. At home, work was often mixed with daily family life and hobbies. Whether it was more peaceful at home or in the office depended on whether the person was at home alone or with family members.

Participants often commented that the photos reflected calmness, proximity, and warmth. In some cases, photos of dogs or photos with outdoor views were described as comforting. The comments also included expressions of aesthetic pleasure and prerequisites for relaxation that related to beautiful places, things and indoor plants, views, forests, snow, sun, nature, companionship, and objects that included water, fire, air, and soil. All these comments were related to supporting health. Further, colleagues were often mentioned for their inspiring, motivating, and refreshing influence – which was an important contribution, as the participants were not allowed to take photos of other people.
