# Supplementary material for: Framework for Health-Promoting Environments for Office Workers: Photovoice Study
Source: JMIR Form Res. 2026 Jun 29;10:e90712. doi: 10.2196/90712 (PMC13313410; doi:10.2196/90712)
Supplement: Multimedia Appendix 2 [file formative-v10-e90712-s002.docx]

This is a Multimedia Appendix to a full manuscript published in JMIR Formative Research. For full copyright and citation information see doi:[**10.2196/90712**](https://doi.org/10.2196/90712).

**Questions on office work developed* based on the findings from the present study.**

A) How much of your working time do you work in an office environment? (Office environment refers to the room or premises your employer offers.)

| At present | Before the COVID-19 pandemic |
| --- | --- |
| ☐ 5 days a week or more [1]  ☐ 4 days a week [2]  ☐ 2–3 days a week [3]  ☐ 1 day a week [4]  ☐ Half a day a week [5]  ☐ No time at all [6]  ☐ Don’t know / Don’t wish to answer [998] | ☐ 5 days a week or more [1]  ☐ 4 days a week [2]  ☐ 2–3 days a week [3]  ☐ 1 day a week [4]  ☐ Half a day a week [5]  ☐ No time at all [6]  ☐ Don’t know / Don’t wish to answer [998] |

A_a) What type of office environment do you work in? Mark only one answer. (Office environment refers to the room or premises your employer offers.)

| At present | Before the COVID-19 pandemic |
| --- | --- |
| ☐ My own room [1]  ☐ My own desk in a room for 2–3 people [2]  ☐ My own desk in a room for 4–9 people [3]  ☐ My own desk in a room for 10–24 people [4]  ☐ My own desk in a room for at least 25 people [5]  ☐ I borrow someone else’s desk that is available [6]  ☐ I may choose among non-personal desks/workplaces [7]  ☐ Other type of workplace [8]  ☐ Don’t know / Don’t wish to answer [998] | ☐ My own room [1]  ☐ My own desk in a room for 2–3 people [2]  ☐ My own desk in a room for 4–9 people [3]  ☐ My own desk in a room for 10–24 people [4]  ☐ My own desk in a room for at least 25 people [5]  ☐ I borrow someone else’s desk that is available [6]  ☐ I may choose among non-personal desks/workplaces [7]  ☐ Other type of workplace [8]  ☐ Don’t know / Don’t wish to answer [998] |

A_b) Do you have access to a room for undisturbed work, telephone calls, spontaneous meetings, etc. in your office environment? (Office environment refers to the room or premises your employer offers.)

| At present | Before the COVID-19 pandemic |
| --- | --- |
| ☐ Yes, to a sufficient extent [1]  ☐ Yes, but to a limited extent [2]  ☐ No, not at all [3]  ☐ Don’t know / Don’t wish to answer [998] | ☐ Yes, to a sufficient extent [1]  ☐ Yes, but to a limited extent [2]  ☐ No, not at all [3]  ☐ Don’t know / Don’t wish to answer [998] |

A_c) Do you consider that your office environment allows you to do a good job? (Office environment refers to the room or premises your employer offers.)

| At present | Before the COVID-19 pandemic |
| --- | --- |
| ☐ Yes, to a high degree [1]  ☐ Yes, to a fairly high degree [2]  ☐ Yes, to some degree [3]  ☐ No, not at all [4]  ☐ Don’t know / Don’t wish to answer [998] | ☐ Yes, to a high degree [1]  ☐ Yes, to a fairly high degree [2]  ☐ Yes, to some degree [3]  ☐ No, not at all [4]  ☐ Don’t know / Don’t wish to answer [998] |

B) How often do you perform office work from home?

| At present | Before the COVID-19 pandemic |
| --- | --- |
| ☐ 5 days a week or more [1]  ☐ 4 days a week [2]  ☐ 2–3 days a week [3]  ☐ 1 day a week [4]  ☐ Half a day a week [5]  ☐ No time at all [6]  ☐ Don’t know / Don’t wish to answer [998] | ☐ 5 days a week or more [1]  ☐ 4 days a week [2]  ☐ 2–3 days a week [3]  ☐ 1 day a week [4]  ☐ Half a day a week [5]  ☐ No time at all [6]  ☐ Don’t know / Don’t wish to answer [998] |

B_a) When working from home do you have access to a room for undisturbed work, telephone calls, spontaneous meetings, etc.?

| At present | Before the COVID-19 pandemic |
| --- | --- |
| ☐ Yes, to a sufficient extent [1]  ☐ Yes, but to a limited extent [2]  ☐ No, not at all [3]  ☐ Don’t know / Don’t wish to answer [998] | ☐ Yes, to a sufficient extent [1]  ☐ Yes, but to a limited extent [2]  ☐ No, not at all [3]  ☐ Don’t know / Don’t wish to answer [998] |

B_b) Do you consider that working from home allows you to do a good job?

| At present | Before the COVID-19 pandemic |
| --- | --- |
| ☐ Yes, to a high degree [1]  ☐ Yes, to a fairly high degree [2]  ☐ Yes, to some degree [3]  ☐ No, not at all [4]  ☐ Don’t know / Don’t wish to answer [998] | ☐ Yes, to a high degree [1]  ☐ Yes, to a fairly high degree [2]  ☐ Yes, to some degree [3]  ☐ No, not at all [4]  ☐ Don’t know / Don’t wish to answer [998] |

C) How often do you perform office work at cafés, restaurants, hotel lobbies or similar public places?

| At present | Before the COVID-19 pandemic |
| --- | --- |
| ☐ Every day [1]  ☐ About once a week [2]  ☐ About once a month [3]  ☐ Even more seldom [4]  ☐ Never [5]  ☐ Don’t know / Don’t wish to answer [998] | ☐ Every day [1]  ☐ About once a week [2]  ☐ About once a month [3]  ☐ Even more seldom [4]  ☐ Never [5]  ☐ Don’t know / Don’t wish to answer [998] |

D) How often do you perform office work outdoors? (Office work outdoors refers to for example, focused work, meetings, or phone calls outdoors.)

| At present | Before the COVID-19 pandemic |
| --- | --- |
| ☐ Every day [1]  ☐ About once a week [2]  ☐ About once a month [3]  ☐ Even more seldom [4]  ☐ Never [5]  ☐ Don’t know / Don’t wish to answer [998] | ☐ Every day [1]  ☐ About once a week [2]  ☐ About once a month [3]  ☐ Even more seldom [4]  ☐ Never [5]  ☐ Don’t know / Don’t wish to answer [998] |

*****The development of the questions included revision of existing questions in the Work Environment survey 2019 Sweden [1], and development of new questions to cover office work environments that were not included in the Work Environment survey.

Reference

1. Arbetsmiljöverket (Swedish Work Environment Authority). Arbetsmiljön 2019 [The Work Environment 2019]. Arbetsmiljöstatistik Rapport 2020:2. Available from: https://www.av.se/globalassets/filer/statistik/arbetsmiljon-2019/arbetsmiljostatistik-arbetsmiljon-2019-rapport-2020-2.pdf [cited March 26, 2025].
